# Tumor Immune Microenvironment and Current Status of Immune Checkpoint Inhibitor Therapy in Colorectal Cancer Liver Metastasis

**DOI:** 10.3390/curroncol32090493

**Published:** 2025-09-02

**Authors:** Dandan Cao, Aiping Zhou

**Affiliations:** Department of Medical Oncology, National Cancer Center/National Clinical Research Center for Cancer/Cancer Hospital, Chinese Academy of Medical Sciences and Peking Union Medical College, Beijing 100021, China; cdddoct@pumc.edu.cn

**Keywords:** tumor immune microenvironment, colorectal cancer liver metastasis, immune checkpoint inhibitors

## Abstract

Colorectal cancer is one of the most common malignancies worldwide, with liver metastasis being one of its primary metastatic patterns and a significant cause of death. For most patients with unresectable colorectal cancer liver metastasis, a comprehensive treatment strategy that includes chemotherapy and targeted therapy is the primary therapeutic strategy. However, significant breakthroughs in immune therapy for liver metastasis have yet to be achieved. In this article, we summarize the characteristics of the immune microenvironment in colorectal cancer liver metastases and the mechanisms of immune resistance. Additionally, we compile recent clinical trial results on immune combination strategies and biomarker studies and discuss the prospects for applying immune checkpoint inhibitors in the treatment of colorectal cancer liver metastasis.

## Simple Summary

Colorectal cancer often spreads to the liver, which greatly affects patient survival. While traditional treatments like chemotherapy and targeted drugs are commonly used, the role of immune therapy in liver metastases is still limited. This review explains how the liver’s unique environment makes it harder for the immune system to fight cancer, and why immune treatments that work in other settings are less effective here. We also highlight promising new research combining immune drugs with other therapies, offering hope for better outcomes in the future.

## 1. Epidemiology and Current Treatment Status of Colorectal Cancer Liver Metastasis

According to the 2022 GLOBOCAN database [1], colorectal cancer (CRC) is the third most common malignancy globally and the second leading cause of cancer-related death (9.3%). The liver is the most frequent site of metastasis in CRC patients [2], with up to 50% of them experiencing liver metastasis during the disease course [3]. For patients with resectable CRC liver metastases (CRLMs), surgical resection combined with perioperative systemic therapy is recommended, and the 5-year survival rate ranges from 20 to 50%. Patients with potentially resectable CRLM may be eligible for surgical resection through conversion therapy. For patients unable to undergo surgical resection, the primary goals of systemic treatment are to improve quality of life and prolong survival. Standard chemotherapy regimens include 5-fluorouracil combined with irinotecan (FOLFIRI) and oxaliplatin (FOLFOX) [4]. Anti-VEGF agents (bevacizumab), anti-EGFR agents (cetuximab or panitumumab), and small-molecule antiangiogenic drugs (regorafenib, fruquintinib) play significant roles in the treatment of advanced CRC [5,6,7]. Immunotherapy, especially immune checkpoint inhibitors (ICIs), plays a crucial role in treating deficient mismatch repair (dMMR)/microsatellite instability-high (MSI-H) CRC. However, their efficacy is minimal in mismatch repair proficient (pMMR)/microsatellite stable (MSS) CRC, especially in CRLM patients, where immunotherapy is nearly ineffective. Approximately 10–15% of primary CRC cases are dMMR/MSI-H. Studies indicate that around 77–80% of metastatic tumors, including liver metastases, maintain dMMR/MSI status consistent with the primary tumor [8,9]. Discordance can occur in a minority of cases, potentially due to intratumoral heterogeneity or prior treatments, highlighting that re-evaluation of MMR/MSI status may be warranted in specific clinical scenarios. Understanding the characteristics of the immune microenvironment in patients with CRLM and the mechanisms of immune resistance may help identify strategies to overcome immune resistance in patients with MSS CRC and liver metastasis.

## 2. Liver Metastasis Is Associated with the Poor Efficacy of Immune Checkpoint Inhibitors

Preclinical studies have revealed differences in the efficacy of ICIs between liver and lung metastasis cohorts. Research by Yu et al. demonstrated that liver metastasis can systematically impair the effectiveness of immunotherapy both preclinically in mouse models and clinically in patients, suggesting that the presence of liver metastasis may serve as an independent predictor of ICI efficacy [10]. In their study, a liver metastasis mouse model was generated by establishing primary tumors through subcutaneous injection of MC38 cancer cells and inducing liver metastasis by inoculating the spleen with MC38 cells. The results revealed that mice without liver metastasis responded to ICI treatment, whereas those with liver metastasis consistently did not respond. Repeating this experiment in a lung metastasis model did not yield the same results, indicating that systemic resistance to immunotherapy occurs primarily in liver metastasis models. These findings suggest significant differences in immune activation and suppression between liver and lung metastases, with liver metastasis potentially uniquely inhibiting systemic antitumor immunity.

Several clinical studies have also confirmed that patients with liver metastasis have poorer responses to ICI treatment than those without liver metastasis. A subgroup analysis of a phase II clinical trial exploring the activity of the PD-L1 monoclonal antibody durvalumab combined with the MEK inhibitor trametinib in MSS metastatic CRC (mCRC) revealed that lung metastasis patients benefited significantly more from combination therapy than liver metastasis patients did [11]. Additionally, an analysis of a subgroup of patients from the REGONIVO trial, which evaluated regorafenib combined with nivolumab in mCRC patients who had failed second-line chemotherapy, revealed that patients with lung metastasis had a higher objective response rate (ORR) of 50.0% (8/16) than liver metastasis patients did, who achieved 15.4% (2/13) [12]. In another study aimed at investigating whether liver metastasis could serve as a marker for ICI resistance in advanced CRC, dual immunotherapy with durvalumab and tremelimumab yielded a disease control rate (DCR) of 49% (90% CI: 36–62%) in patients without liver metastasis. In comparison, the DCR of patients with liver metastasis was only 14% (90% CI: 6–38%; *p* = 0.03). Additionally, data from a multivariate analysis indicated that patients without liver metastasis had significantly improved overall survival (OS) and progression-free survival (PFS) compared with patients with liver metastasis, further suggesting that the presence of liver metastasis is associated with poorer clinical outcomes and adverse results from ICI treatment in advanced CRC patients [13]. Consistent with findings in colorectal cancer, the negative impact of liver metastases on ICI efficacy has also been observed in other malignancies. For example, a recent meta-analysis of 24 randomized controlled trials in lung cancer demonstrated that patients with liver metastases derived significantly less benefit from ICIs compared with those without (pooled OS HR 0.83 vs. 0.73), highlighting the organ-specific immunosuppressive role of the liver across different cancer types [14]. Overall, these studies have consistently revealed a correlation between the presence of liver metastasis and the poor efficacy of ICIs.

Interestingly, hepatocellular carcinoma (HCC), another primary hepatic malignancy, has demonstrated substantial benefit from ICIs, in contrast to the limited efficacy observed in CRLM. Several pivotal phase III trials have reported the efficacy of ICIs in HCC. For instance, IMbrave150 showed that atezolizumab plus bevacizumab significantly improved both OS and PFS [15]. Collectively, these findings suggest that the discrepancy between CRLM and HCC may arise from fundamental differences in the tumor immune microenvironment between primary hepatic malignancies and metastatic lesions. Despite certain limitations, such as the limited number of liver metastasis patients and the potential lack of representativeness in the experimental data for specific ICIs, the impact of liver metastasis on the efficacy of ICIs is a topic worthy of further exploration.

## 3. Liver Metastasis and the Immune Microenvironment

### 3.1. Immune Microenvironment of Liver Metastases

**Figure 1:**
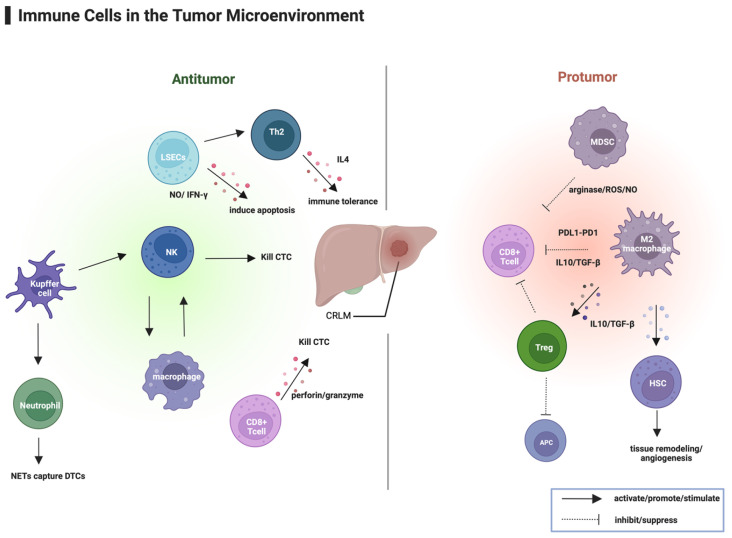
Immune cells in the tumor microenvironment of colorectal liver metastases (CRLM). Immune cells exert both antitumor and protumor effects within the CRLM microenvironment. Legend note: LSECs, NK cells, Kupffer cells: support cytotoxicity against disseminated tumor cells; enhanced by STING agonists or NK cell–based therapies. Neutrophils (NETs): promote metastatic seeding; inhibited by NET-targeted therapies or IL-8 blockade. M2 macrophages/TAMs: mediate immune suppression (IL-10, TGF-β); targeted by TGF-β blockade or PD-L1 inhibitors. Tregs: suppress effector T cells; reduced by Treg depletion or anti-CTLA-4 antibodies. MDSCs: impair T-cell proliferation; targeted by MDSC-directed inhibitors. HSCs: promote angiogenesis and ECM remodeling when activated by M2 macrophages; inhibited by anti-angiogenic therapy (e.g., bevacizumab). CD8+ T cells: undergo exhaustion via PD-1/PD-L1; reinvigorated by PD-1/PD-L1 blockade. Created in BioRender. Cao, D. (2025) https://BioRender.com/bv1405q (accessed on 1 September 2025).

The tumor microenvironment (TME) comprises the surroundings of tumor cells, including tumor cells themselves; various immune cells, such as macrophages, neutrophils, T lymphocytes, and dendritic cells; and stromal cells, such as tumor-associated fibroblasts and endothelial cells, along with the extracellular matrix. The TME plays a critical role in tumor invasion and metastasis, as illustrated in Figure 1, which depicts the immune cells within the tumor microenvironment of CRLM. Before the development of CRC liver metastasis, hepatocytes secrete various factors to recruit or activate immune and stromal cells within the liver, forming a premetastatic niche [16]. In the early stage of CRLM, various immune cells contribute to the destruction and elimination of tumor cells through phagocytosis and cytotoxic functions. Liver sinusoidal endothelial cells (LSECs) enhance disseminated tumor cell (DTC) retention by expressing adhesion molecules and releasing nitric oxide (NO) and interferon-gamma (IFN-γ), which can induce apoptosis in tumor cells entering the sinusoids. Moreover, LSECs in the liver can inhibit Th1 cells expressing IFN-γ and promote Th2 cells expressing IL-4, ultimately contributing to immune tolerance in the liver [17]. Kupffer cells not only directly phagocytose tumor cells but also release multiple cytokines and chemokines to activate other innate immune response cells, such as natural killer (NK) cells and neutrophils [18]. NK cells exert antitumor effects by directly killing target cells, whereas STING signaling in macrophages enhances NK cell activity through NLRP3 activation and cytokine secretion (e.g., IL-1β, IL-18), which upregulate 4-1BB on NK cells and 4-1BBL on macrophages, forming a positive feedback loop that enhances NK cell activity [19]. However, during tumor progression, these liver-resident cells can also contribute to immune tolerance by secreting the anti-inflammatory cytokines such as IL-10 and transforming growth factor-beta (TGF-β), and expressing PD-L1, which hinders T-cell activation [20]. M2-type KCs secrete TGF-β, which not only inhibits T-cell activity and induces the generation of Tregs but also activates hepatic stellate cells (HSCs), contributing to extracellular matrix remodeling, angiogenesis, and tumor progression [21]. Tregs can inhibit the maturation of antigen-presenting cells (APCs) and clear effector T cells and APCs, thereby reducing the cytotoxic function of CTLs [17,22]. Myeloid-derived suppressor cells (MDSCs), which are enriched in CRLM, weaken antitumor immunity by inhibiting T-cell proliferation, disrupting the T-cell receptor (TCR) structure, and inducing T-cell apoptosis [17,23]. Tumor-associated neutrophils (TANs) also contribute to liver metastasis by extruding chromatin fibers to form neutrophil extracellular traps (NETs), which trap CRC cells in the liver. NET-induced IL-8 expression enhances CRC cell proliferation and invasion, and IL-8 further activates neutrophils to generate more NETs, establishing a positive feedback loop that promotes CRC liver metastasis [24]. These immune suppressive mechanisms create a favorable environment for tumor growth and progression.

From a histopathological perspective, patients with resected CRLM exhibiting only the desmoplastic histopathological growth pattern (dHGP) have superior survival, which may be attributed to the peritumoral and intratumoral enrichment of cytotoxic CD8+ T cells and a higher CD8+/CD4+ T-cell ratio. MSI is more frequently observed in colorectal liver metastases with a dHGP compared with non-desmoplastic metastases (14.6% vs. 3.6%, *p* = 0.01) [25]. This enrichment may partly explain the enhanced immune infiltration and favorable responses to ICIs seen in dHGP tumors [26]. Recent studies utilizing single-cell RNA sequencing and spatial transcriptomics to characterize CRLM have identified highly metabolically activated immune suppressive cells, such as MRC1+ CCL18+ M2-like macrophages, at metastatic sites [27]. Another study revealed that a specific CD274+ (PD-L1+) CD206+ (MRC1+) macrophage subgroup could predict poor prognosis in CRC patients. This tumor-associated macrophage (TAM) subgroup promotes tumor growth by inhibiting CD8+ T-cell activity and fostering an immunosuppressive microenvironment [28]. These findings suggest that the immune microenvironment in CRLM patients contains more immunosuppressive components than the microenvironment in matched patients, which may partially explain the poor responsiveness of liver metastases to ICIs. Additionally, the increased presence of CD206+ TAMs highlights a promising immunotherapeutic target. Combination therapies targeting immune cells with immune checkpoint inhibitors may help reverse the immunosuppressive environment and improve immunotherapy outcomes.

### 3.2. Systemic Immune Suppression Induced by Liver Metastasis

The mechanism of systemic immune suppression may be related to macrophage-mediated T-cell elimination. In preclinical liver models, activated antigen-specific Fas+CD8+ T cells undergo apoptosis upon interaction with FasL+CD11b+ F4/80+ monocytic macrophages, leading to systemic immune desertification. This observation aligns with the reduced peripheral T-cell counts and diminished diversity and functionality of tumor-infiltrating T cells in patients with liver metastasis [10]. In another study, liver tumor-associated Tregs were reported to modulate antigen-specific immunity in distant organs. Treg depletion or inactivation can reverse tumor-associated systemic immune suppression, suggesting that Tregs may regulate systemic immunity and responses to immunotherapy in liver metastases [29]. Additionally, preclinical studies have shown a significant reduction in activated T cells and dendritic cells in the immune microenvironment of liver metastases compared with those in subcutaneous tumor metastases. Further research demonstrated that a treatment strategy combining FLT3 ligands with ICIs could increase dendritic cell infiltration in liver metastases and prolong survival in mice. These findings indicate that dendritic cells may increase the efficacy of immunotherapy in pMMR CRC [30]. These studies collectively highlight the negative regulation of systemic immunity by liver metastases. Targeting this process may improve the effectiveness of ICIs.

### 3.3. Influence of the Gut Microbiota on the Immune Microenvironment of Liver Metastases

The gut microbiota influences tumor progression and the TME through various mechanisms. For example, Fusobacterium nucleatum promotes CRC metastasis by reducing METTL3-mediated m6A modification, which further influences tumor transcription [31]. Dysbiosis of the gut microbiota has a significant effect on CRC metastasis, particularly through remodeling of the immune microenvironment at the metastatic site. For example, Escherichia coli C17 can disrupt the gut vascular barrier (GVB), establishing a premetastatic immune microenvironment in the liver to facilitate colorectal cancer liver metastasis [32]. Fusobacterium nucleatum activates the NF-κB/miR-1322 axis to upregulate CCL20, promoting macrophage infiltration and M2 polarization and thus enhancing CRC metastasis [33]. In addition, Fusobacterium nucleatum increases the levels of proinflammatory cytokines such as TNF-α, IL-6, and IL-17A, which promote CRC metastasis and decrease the cytotoxic activity of CD8+ T cells, reducing antitumor immunity. Furthermore, Fusobacterium nucleatum diminishes innate immune responses by decreasing the levels of NK cells and T cells while activating Tregs, thus creating an environment favorable for tumor progression and immune evasion within the liver [34].

Recent studies have also shown that Fusobacterium nucleatum-derived succinic acid may interfere with the cGAS-IFN-β pathway, a key player in CD8+ T-cell trafficking to the tumor microenvironment, thereby diminishing the antitumor response. Additionally, fecal microbiota transplantation (FMT) from low-Fusobacterium nucleatum responders has been shown to restore sensitivity to anti-PD1 immunotherapy in mice, indicating that the gut microbiota plays a significant role in modulating tumor resistance to immunotherapy. These findings highlight the potential of targeting dysbiosis in mCRC patients as a promising therapeutic strategy [35]. In mCRC, a study involving FMT combined with regorafenib and toripalimab demonstrated that in refractory MSS metastatic colorectal cancer patients, the median progression-free survival (PFS) was 9.6 months, and the median overall survival (OS) was 13.7 months, with high survival rates and manageable safety [36]. This result provides a valuable new therapeutic option for this patient population.

### 3.4. Unique Immune Cell Composition and Spatial Distribution in the TME of Liver Metastases

Given that liver metastases significantly suppress immune checkpoint inhibitor efficacy, several studies have utilized next-generation sequencing and spatial transcriptomics to compare the immune microenvironments of primary and liver metastatic tumors in patients with CRC. One study evaluated the expression patterns of TAM populations in CRLM, CRC patients and nonmetastatic HCC, reporting that SPP1+ TAMs were enriched in liver metastases, with levels even higher than those in matched primary CRC tumors, despite their absence in nonmetastatic HCC. Subsequent survival analyses using The Cancer Genome Atlas (TCGA) datasets revealed that higher levels of SPP1, or the signature of SPP1+ TAMs, were associated with worse OS in treatment-naive patients. These findings suggest that SPP1+ macrophages are predominant in liver metastases, indicating their prometastatic role [37]. Single-cell RNA sequencing (scRNA-seq) of the TME in MSS CRC liver metastases revealed that TME-specific SPP1+ macrophages and fibroblasts, which are in close spatial proximity, expressed complementary ligand–receptor pairs, suggesting the potential for reciprocal regulation of gene expression programs and intercellular communication. Additionally, the TME of MSS CRC liver metastases is characterized by a lack of dysfunctional CD8+ T cells and an increased presence of regulatory T cells, indicating an immunosuppressive environment [38]. Other studies revealed distinct immune cell compositions and spatial distributions within liver metastases. In their investigation of the spatial distribution differences in various types of TAMs in primary and metastatic sites through colocalization of CD68 and CD163, He et al. reported the reorganization of TAMs in liver metastases. Compared with those in primary tumors, immune suppressive cells, such as CD68-CD163+ macrophages, are present at high levels in the peritumoral (PT), tumor invasive front (TF), and tumor center (TC) regions of liver metastases [39]. Zhou et al. compared the density of infiltrating immune cells (CD3+ cells, CD8+ cells, CD11b+ cells, CD11c+ cells, and CD33+ cells) in primary and liver metastatic tumors and reported that the number of immune suppressive cells, such as CD33+ cells and CD8+ cells (but not CD8+ T cells), was significantly higher in liver metastases than in primary tumors. This may partially explain the intrahepatic immune suppression and poor efficacy of immunotherapy [40]. Moreover, different immune cell subpopulation abundances and spatial distributions display organ-specific characteristics at various metastatic sites. In contrast to that of liver metastases, the TC region of lung metastases has significantly higher T-cell and B-cell levels, a higher density of antigen-presenting cells and their interactions with T-cells, and more extensive lymphoid aggregates. Lung metastases, which are more immunocompetent, express higher STING levels in the tumor core. The cGAS-STING pathway, which facilitates tumor-immune cell crosstalk by activating antigen-presenting cells such as dendritic cells through tumor-derived DNA, triggers immune responses, including type I IFN secretion, tertiary lymphoid structure formation, and enhanced antitumor responses by T cells and NK cells. Consistent with these findings, lung metastases present higher levels of T-cell activation markers (CD27, CD44, ICOS, 4-1BB, and CD45RO) than liver metastases do, indicating a more active immune microenvironment in lung metastases [41]. Similar findings have been reported in non-small cell lung cancer patients. CD8+ cell fractions were significantly lower (*p* = 0.036) in the LM group than in the paired pleural group, with reduced CD8+ T-cell activation and NK cell cytotoxicity in LMs, which may explain their poorer response to immunotherapy [42]. The unique immune cell composition and spatial distribution in the TME of liver metastases may partially explain their “cold” immune characteristics, although the specific mechanisms require further investigation.

Currently, strategies targeting immunosuppressive myeloid cells and Treg modulation are under investigation in early-phase clinical trials. Microbiota-targeted interventions such as FMT have entered clinical studies and shown preliminary efficacy. In contrast, biomarker development based on spatial transcriptomics and certain rational combination strategies remain at the preclinical or exploratory stages, requiring further validation in prospective clinical studies before potential translation into routine practice.

## 4. ICIs for Advanced Colorectal Cancer Liver Metastasis

### 4.1. Preclinical Research Targeting the Immune Microenvironment of CRLM

Given the unique composition of the immune microenvironment in liver metastases and the systemic immune suppression caused by liver metastasis, several preclinical studies have explored therapeutic strategies while attempting to elucidate the underlying mechanisms involved. In preclinical models, liver metastases have been shown to draw off CD8+ T cells and induce apoptosis through macrophage interactions, leading to immune therapy resistance. Directional radiotherapy targeting the liver can eliminate immunosuppressive liver macrophages, thereby increasing T-cell survival and reducing their recruitment into the liver. On this basis, a combined treatment strategy involving liver-directed radiotherapy and ICIs has been proposed to enhance systemic antitumor immunity and achieve significantly better outcomes [10]. Interleukin-10 (IL-10), which is produced primarily by macrophages, T cells, and tumor cells in CRLM, plays a crucial role in immune suppression. In human tumor slice cultures (TSCs) from patients with CRLM, the addition of anti-IL-10 neutralizing antibodies significantly increased the proportion of CD8+ T cells and HLA-DR expression on macrophages, resulting in a 1.8-fold increase in T-cell-mediated tumor cell killing. These findings indicate that blocking IL-10 could partially overcome the immunosuppressive TME and reactivate endogenous antitumor immunity. These findings suggest that targeting IL-10 may be a promising systemic therapy for MSS CRLM patients resistant to ICIs [43]. In one study, the expression of LAG3 on tumor-infiltrating lymphocytes (TILs) was reported to be upregulated after microwave ablation (MWA) treatment. Combining LAG3 inhibitors with MWA significantly promoted the proliferation and antitumor function of CD8+ TILs, delaying tumor progression and extending survival in an MC38 tumor model [44]. In another study, ferroptosis was reported to be useful in the treatment of primary liver tumors and liver metastases from other types of cancer. Inducing ferroptosis by knocking out the Gpx4 gene and blocking MDSCs and neutrophils significantly enhanced the response of CRC liver metastases and primary hepatocellular carcinoma to ICIs. Combining ferroptosis induction with ICIs and MDSC blockade may represent an effective anticancer approach for treating hepatocellular carcinoma and CRLM [45]. Notably, this combined approach showed efficacy in liver metastases or CRC liver metastases but demonstrated resistance in primary CRC, potentially due to the unique liver microenvironment [46]. However, these findings are primarily from preclinical studies, and their conclusions and feasibility await further validation in subsequent clinical trials.

### 4.2. Current Status of Immunotherapy in mCRC Liver Metastasis

Although there are currently no clinical studies specifically targeting immunotherapy combinations for patients with CRLM, several clinical trials in nonselective mCRC populations have explored various ICI combination strategies. The results of these subgroup analyses offer valuable guidance for future research. In MSI-H/dMMR mCRC, different TMB cutoff values have been shown to predict disease progression and treatment benefit. For MSS mCRC in first-line treatment, although the results from subgroup analyses regarding liver metastasis are inconsistent among studies, the CheckMate 9X8 phase II study demonstrated that compared with standard-of-care (SOC) alone, the CMS1 and CMS3 subgroups presented improved PFS with nivolumab combined with SOC [47]. The AtezoTRIBE phase II study revealed that patients with high Immunoscores and high TMBs experienced survival benefits [48]. For later-line treatment in MSS mCRC, the CO.26 study suggested that elevated plasma TMB can identify patients most likely to benefit from combined immune checkpoint inhibition [49]. These findings highlight the potential of refining subgroup selection based on these biomarkers to better tailor immunotherapy strategies, particularly for MSS mCRC patients with liver metastasis.

#### 4.2.1. Research on ICIs in MSI-H/dMMR mCRC

In populations with advanced MSI-H/dMMR mCRC, immunotherapy has shown significant benefits over chemotherapy. In the KEYNOTE-177 study, the pembrolizumab group showed a median PFS of 16.5 months, compared to 8.2 months in the chemotherapy group (hazard ratio 0.60, 95% CI 0.45–0.80), which supports its recommendation as a first-line standard treatment for MSI-H/dMMR mCRC [50]. In the Phase II CheckMate-142 study, patients with dMMR/MSI-H mCRC were reported to remarkably benefit from first-line treatment with nivolumab combined with ipilimumab [51], with a response rate of 69% (95% CI, 53 to 82) and a disease control rate of 84% (95% CI, 70.5 to 93.5). Currently, the CSCO guidelines recommend pembrolizumab and nivolumab combined with ipilimumab as the palliative first-line treatment for dMMR/MSI-H mCRC. Although dMMR/MSI-H mCRC patients generally have a relatively high TMB, specific values can vary. A study revealed that different TMB cutoff values can predict disease progression and treatment benefit in patients treated with ICIs. A lower TMB is correlated with earlier disease progression, whereas patients with a higher TMB may benefit the most from enhanced CTLA-4/PD-1 therapy [52].

#### 4.2.2. Research on ICIs in MSS mCRC First-Line Treatment

In the first-line treatment of MSS mCRC, attempts to combine single-agent immunotherapy with standard regimens have generally not demonstrated significant benefits across most studies. In the CheckMate 9X8 phase II study, nivolumab combined with standard-of-care (SOC) treatment (mFOLFOX6 plus bevacizumab) was compared with SOC treatment in mCRC patients, and the results indicated that the median progression-free survival (mPFS) time was 11.9 months in both groups. In the liver metastasis subgroup (*n* = 142), the median PFS was 10.2 months in the nivolumab plus SOC group, compared to 11.4 months in the SOC group (unstratified hazard ratio 0.80, 95% CI: 0.49–1.30). No improvement in PFS was observed with the addition of nivolumab to SOC treatment in this subgroup. These findings suggest that combining PD-1 monoclonal antibodies with chemotherapy does not enhance efficacy in the overall population of pMMR mCRC patients or in those with liver metastases. Subgroup analysis revealed that in the baseline CMS1 and CMS3 subgroups of MSS/pMMR patients, nivolumab combined with SOC was associated with better PFS than SOC alone after 12 months [47]. In the AtezoTRIBE phase II study, atezolizumab combined with SOC treatment (FOLFOXIRI plus bevacizumab) was compared to SOC alone in the first-line treatment of mCRC patients, and the results showed that there was no significant difference in median overall survival (mOS) between the two groups in the pMMR population. However, subgroup analysis revealed that patients with high immune cell scores and a high tumor mutational burden (TMB) experienced significant survival benefits, suggesting that the immune cell score and TMB may be potential biomarkers for MSS mCRC patients receiving first-line combination immunotherapy [48]. In the ASTRUM-015 study, the safety and efficacy of first-line treatment in mCRC patients, namely the standard treatment with bevacizumab plus XELOX versus the addition of the PD-1 monoclonal antibody serplulimab, were compared, and the results indicated that the mPFS in the combination immunotherapy group was superior to that in the control group (17.2 vs. 10.7 months; stratified HR 0.60, 95% CI 0.31–1.14), confirming that the addition of serplulimab to targeted therapy and chemotherapy has stronger antitumor activity [53]. According to the follow-up data presented in a poster at ASCO 2024, by the data cutoff of 15 December 2023, the combination immunotherapy group demonstrated sustained benefits in terms of mPFS (16.8 vs. 10.7 months; stratified HR 0.62, 95% CI 0.33–1.12) and mOS (not reached vs. 21.2 months; stratified HR 0.75, 95% CI 0.43–1.30). In key subgroups, including MSS patients (not reached vs. 21.2 months; stratified HR 0.73, 95% CI 0.39–1.37), patients with KRAS mutations (21.9 vs. 18.2 months; unstratified HR 0.72, 95% CI 0.37–1.39), and liver metastasis patients (not reached vs. 20.2 months; unstratified HR 0.75, 95% CI 0.40–1.41), the serplulimab group had a favorable trend toward improved OS [54]. The above studies indicate that with an extended follow-up time, the survival benefits of the combination with serplulimab are maintained in the first-line treatment of mCRC, while safety remains manageable. These findings suggest that the results of combining immunotherapy with chemotherapy in MSS mCRC are inconsistent, but the ASTRUM-015 study highlights the potential of adding serplulimab to standard therapy, demonstrating significant benefits in mPFS and mOS, particularly in subgroups such as those with liver metastases and KRAS mutations.

Initial attempts to combine dual immunotherapy with standard regimens have shown promise. In the MEDITREME study, the combination strategy of dual immunotherapy with chemotherapy was explored, with durvalumab combined with tremelimumab and mFOLFOX6 investigated as the first-line treatment for RAS-mutant mCRC, and the Phase 1b results indicated good safety. In the MSS cohort (*n* = 48), no significant differences in PFS were observed between the liver metastasis subgroup and the non-liver metastasis subgroup. In MSS mCRC patients, the PFS rate after 3 months of treatment with this combination regimen was 90.7%, the overall response rate (ORR) was 64.5%, and the mPFS was 8.2 months, with the OS not yet reached [55]. This study revealed that in MSS mCRC patients, dual immunotherapy combined with mFOLFOX6 as a first-line treatment regimen has good tolerability and clinical activity, with similar efficacy observed in patients with and without liver metastases. Compared with monotherapy, dual immunotherapy combined with standard regimens may have more significant potential in the first-line treatment of MSS mCRC, although further research is needed for validation. However, current data on combination regimens involving ICIs in first-line treatment are insufficient, and subgroup analysis results for liver metastases are inconsistent, making it difficult to form clear conclusions, so further investigation is required.

#### 4.2.3. Research on ICIs in MSS mCRC Later-Line Treatment

The efficacy of ICIs combined with tyrosine kinase inhibitors (TKIs), such as lenvatinib and regorafenib, for later-line treatment of MSS mCRC has been explored in multiple studies, with most showing no advantages in the liver metastasis subgroup. In a phase III trial, namely, LEAP-017, the combination of lenvatinib and pembrolizumab was assessed in previously treated MSS/pMMR mCRC patients, and the results revealed trends toward improved OS, PFS, and ORR in the combination group compared with the SOC group. However, these findings did not reach the predefined threshold of significance. Notably, subgroup analysis revealed that patients without liver metastases experienced more significant benefits from the combination of lenvatinib and pembrolizumab (HR 0.65, 95% CI: 0.42 to 0.99 months), whereas no significant difference was observed in the liver metastasis subgroup. These findings suggest that, compared with patients with SOC, patients without liver metastases are more likely to benefit from this combination therapy. However, the sample size was insufficient for robust comparisons of the liver and non-liver metastasis subgroups [56]. In the REGONIVO phase Ib trial, the efficacy and safety of regorafenib combined with nivolumab was explored in mCRC patients in which second-line chemotherapy had failed, and the results revealed an ORR of 36% and a PFS of 7.9 months, indicating that ICIs combined with TKIs have some antitumor activity with manageable safety. However, further research in larger cohorts is needed. The aim in the RIN study was to investigate the addition of the CTLA-4 antibody ipilimumab to the REGONIVO regimen to enhance efficacy. In the RP2D cohort of the RIN protocol, the ORR and disease control rate (DCR) were 27.6% and 62.1%, respectively, with median PFS and OS times of 4 months (95% CI: 3 to 9 months) and 20 months, respectively (95% CI: 9 months to not reach), and these therapeutic effects were encouraging. However, subgroup analyses revealed that the ORRs were 0% for liver metastasis patients and 36.4% for non-liver metastasis patients, suggesting that this regimen did not have advantages in the liver metastasis subgroup, highlighting the unique challenges posed by the liver metastasis microenvironment [57]. The phase 2 CO.26 study explored tremelimumab and durvalumab combined with best supportive care (BSC) versus BSC in advanced colorectal cancer. In MSS patients, OS was significantly improved with the combination (HR, 0.66; 90% CI, 0.49–0.89; *p* = 0.02). The greatest benefit was observed in MSS patients with a plasma TMB ≥ 28 variants per megabase (HR, 0.34; 90% CI, 0.18–0.63; *p* = 0.004). These findings suggest that combined immune checkpoint inhibition with durvalumab and tremelimumab may improve OS and that elevated plasma TMB may identify patients who are most likely to benefit [49]. In another phase I/Ib clinical trial of QL1706 (PSB205), the safety, pharmacokinetics, pharmacodynamics, and preliminary efficacy of this dual-function monoclonal antibody (anti-PD-1 IgG4 and anti-CTLA-4 IgG1) were investigated in patients with advanced solid tumors. The results showed that QL1706 was well-tolerated in patients with refractory colorectal cancer in later-line treatment, with an objective response rate (ORR) of 7.4% and a disease control rate (DCR) of 25.9%, demonstrating some antitumor activity. Although most early studies suggest that immune checkpoint inhibitors have limited efficacy in pMMR colorectal cancer, these studies suggest that combination therapy with different immune checkpoints may have the potential to improve treatment efficacy [58].

Explorations of combining epigenetic inhibitors with targeted therapies are also ongoing. In the CAPability-01 study, the efficacy of the HDAC inhibitor chidamide combined with sintilimab with or without bevacizumab was investigated in MSS/pMMR mCRC patients with previous failure of standard chemotherapy, and the results revealed a median PFS of 7.3 months, an ORR of 44.0%, and a DCR of 72.0%, suggesting that combining epigenetic immune targeting with a three-drug strategy is promising in the treatment of MSS/pMMR mCRC in later lines. The subgroup analysis results indicated that in the triplet therapy arm, the efficacy of treatment in patients with liver metastasis (*n* = 14) was similar to that in patients without liver metastasis (*n* = 11), with a median PFS of 7.3 months versus 6.7 months (*p* = 0.617). Among the 26 patients with liver metastasis, those receiving triplet therapy (*n* = 14) had a longer median PFS (7.3 months vs. 1.4 months, *p* = 0.001), higher ORR (50.0% vs. 8.3%, *p* = 0.036), and higher DCR (71.4% vs. 8.3%, *p* = 0.002) than those receiving doublet therapy (*n* = 12) [59]. These results suggest that triplet therapy, which adds bevacizumab to the doublet regimen, may improve treatment outcomes in colorectal cancer, particularly those with liver metastasis.

A summary of key clinical trials investigating immune checkpoint inhibitors in metastatic colorectal cancer, including study designs, patient populations, and main results, is provided in Table 1. In summary, while exploration of single-agent immunotherapy combined with TKIs has not yet achieved significant breakthroughs in later-line treatment for MSS mCRC, further refinement of MSS mCRC subgroups or exploration of other immunotherapy-based combinations remains essential.

## 5. Immunoscore as a Promising Biomarker for mCRC

The exploration of new biomarkers, such as the Immunoscore, which assesses immune cell infiltration and the immune status of the TME, is ongoing to identify populations that are more likely to benefit. The prognostic value of the immunoscore as a supplement to the TNM staging system in predicting the time to recurrence in patients with resectable colorectal cancer has been validated. In a large international study initiated by the Society for Immunotherapy of Cancer (SITC), involving 2681 patients, the Immunoscore (47%) outperformed other clinical parameters, including AJCC/UICC TNM staging (28%), grade of differentiation (15%), sex (<3%), and MSI status, in predicting recurrence risk. In this study, the Immunoscore was defined as the evaluation of the densities of CD3+ and CD8+ T cells in both the tumor core and invasive margin, with the mean of four density percentiles assessed by digital pathology [60]. The 2020 ESMO Clinical Practice Guidelines recommend the Immunoscore as a pathological assessment method for predicting postoperative recurrence risk and guiding adjuvant treatment strategies in stage II–III colon cancer patients [61].

While data on the Immunoscore in CRLM patients are lacking, analyses of mCRC patients suggest its potential predictive value. The results of the AtezoTRIBE trial indicate that the Immunoscore may be a reliable predictor of benefit from the addition of atezolizumab to first-line FOLFOXIRI plus bevacizumab [48]. In this study, the Immunoscore IC was defined as the evaluation of PD-L1 and CD8+ T-cell densities, as well as their spatial proximity within a single tissue section, as assessed via image analysis tools. In the multivariate analysis, high Immunoscore IC tumors were independently associated with a progression-free survival benefit in the atezolizumab group (HR 0.19, 95% CI 0.07–0.5). The updated results of the AtezoTRIBE study indicate that, in the pMMR group, patients with high Immunoscore IC tumors derived a greater OS benefit from atezolizumab treatment than did those with low Immunoscore IC tumors (HR 0.25, 95% CI 0.08–0.76 vs. HR 1.0, 95% CI 0.57–1.75) [62]. A phase III trial (NCT06733038) investigating FOLFOXIRI plus bevacizumab with or without atezolizumab as a first-line treatment for patients with initially unresectable, previously untreated pMMR and Immunoscore IC-high mCRC is ongoing, which may further validate these observations.

Furthermore, different studies have defined the Immunoscore in various ways. Given the complexity of the immune microenvironment, in some studies, surface markers of tumor-infiltrating cells (e.g., CD3+, CD8+, CD11b+, CD11c+, and CD33+), PD-L1, PD-1, and VEGFR have been incorporated into the immunoscore to better reflect this complexity [39,40]. Future efforts are needed to establish a standardized scoring methodology and operational guidelines to make the Immunoscore a more practical and valuable biomarker. Studies on the use of the Immunoscore for selecting patients with advanced colorectal cancer, as well as the development of more comprehensive and effective immune-based scoring systems, are still ongoing.

## 6. Summary and Prospects

Owing to the unique immune composition and spatial distribution of the TME in liver metastases, along with systemic immune suppression, CRLM presents a “cold” tumor immune microenvironment. Strategies to overcome these problems include reducing T-cell apoptosis, enhancing T-cell infiltration, antagonizing immunosuppressive cytokines, and upregulating immune checkpoint expression on tumor-infiltrating lymphocytes.

MSS CRC patients exhibit resistance to ICI monotherapy with poor clinical efficacy. However, combining ICIs with chemotherapy and bevacizumab in first-line treatment has shown early potential. Additionally, the combination of sintilimab with chidamide and bevacizumab in later-line treatment holds promise. Although no dedicated immunotherapy trials have specifically involved patients with CRLM, studies in nonselective mCRC populations have offered some insights. Subgroup analyses of liver metastases in first-line ICI combination treatments have shown inconsistent results, while no advantages noted in later-line treatments with a TKI plus an ICI. Several ICI-based large-scale phase III trials are ongoing, and the results will determine whether the addition of an ICI would improve efficacy. Given the unique immune microenvironment of liver metastases, exploring epigenetic inhibitors, functionally enhanced CAR-T or TCR-T-cell therapies, and other immune-based combinations may offer potential solutions to overcome immune tolerance. A critical challenge is identifying biomarkers to select MSS mCRC patients who are likely to benefit from ICI therapy. Emerging biomarkers, including Immunoscore, ctDNA, and maxVAF, necessitate additional investigation to validate their role in guiding precision treatment for CRC.

## Figures and Tables

**Table 1:** Summary of Clinical Trials for ICIs in mCRC.

Key Trial/Clinical Trial Information	Phase	Design (*N*)	Treatment Line Number	Subject	Intervention	Main Results
KEYNOTE-177/NCT02563002	3	307	First-line	MSI-H/dMMR mCRC	Pembrolizumab (*n* = 153) versus chemotherapy (*n* = 154)	mOS: not reached (95% CI: 49.2-not reached) vs. 36.7 m (95% CI: 27.6-not reached)
checkmate-142/NCT02060188	2	45	First-line	MSI-H/dMMR mCRC	Nivolumab plus low-dose ipilimumab	ORR: 69% (95% CI: 53 to 82) DCR: 84% (95% CI: 70.5 to 93.5)
CheckMate 9X8/NCT03414983	2	195	First-line	mCRC	Nivolumab plus SOC versus SOC (mFOLFOX6 + bevacizumab)	PFS did not meet the prespecified threshold for statistical significance.
AtezoTRIBE/NCT03721653	2	218	First-line	mCRC	Atezolizumab combined with SOC versus SOC (FOLFOXIRI + bevacizumab)	mPFS: 13.1 m (80% CI:12.5–13.8) vs. 11.5 m (10.0–12.6) *p* = 0.012
ASTRUM-015/NCT04547166	2/3	114	First-line	mCRC	Serplulimab plus HLX04 and XELOX group versus placebo plus bevacizumab and XELOX	mPFS: (17.2 vs. 10.7 m; stratified HR 0.60, 95% CI 0.31–1.14) mOS was not reached in either group (stratified HR 0.77, 95% CI: 0.41–1.45)
MEDITREME/NCT03202758	1b/2	57	First-line	RAS-mutated mCRC	Durvalumab + tremelimumab + mFOLFOX6 versus mFOLFOX6	3-month PFS of 90.7% (95% CI: 79.2–96%) ORR: 64.5% mPFS: 8.2 m (95% CI: 5.9–8.6)
LEAP-017/NCT04776148	3	480	Laterline	MSS/pMMR mCRC	Lenvatinib plus pembrolizumab versus SOC	mOS: 9.8 m versus 9.3 m (HR, 0.83 (95% CI, 0.68 to 1.02) *p* = 0.0379
REGONIVO/NCT03406871	Ib	50	Laterline	Advanced Gastric or Colorectal Cancer	Regorafenib plus nivolumab	ORR in gastric and colorectal cancer 44% vs. 36%; mPFS in gastric and colorectal cancer 5.6 m vs. 7.9 m
RIN/NCT04362839	1	39	Laterline	MSS/pMMR mCRC the RP2D cohort	Regorafenib, ipilimumab, and nivolumab	ORR: 27.6%; PFS: 4 m (IQR, 2–9 m) OS: 20 m (IQR, 7 m to NE)
QL1706/NCT05576272, NCT05179317	Ib	27	Laterline	Advanced Colorectal Cancer	QL1706	ORR: 7.4% (95% CI: 0.9 to 24.3) DCR: 25.9% (95% CI: 11.1 to 46.3)
CAPability-01/NCT04724239	2	48	Laterline	MSS/pMMR mCRC	Sintilimab, chidamide with or without bevacizumab (the triplet arm vs. the doublet arm)	18wPFS rate (64.0% vs. 21.7%, *p* = 0.003) ORR (44.0% vs. 13.0%, *p* = 0.027)

## Abbreviations

The following abbreviations are used in this manuscript:

CRC	Colorectal Cancer
CRLMs	Colorectal Cancer Liver Metastases
ICIs	Immune Checkpoint Inhibitors
dMMR	Deficient Mismatch Repair
MSI-H	Microsatellite Instability-High
pMMR	Mismatch Repair Proficient
MSS	Microsatellite Stable
mCRC	Metastatic Colorectal Cancer
ORR	Objective Response Rate
DCR	Disease Control Rate
OS	Overall Survival
PFS	Progression-Free Survival
mPFS	Median Progression-Free Survival
TME	Tumor Microenvironment
LSECs	Liver Sinusoidal Endothelial Cells
DTCs	Disseminated Tumor Cells
NO	Nitric Oxide
IFN-γ	Interferon-Gamma
NK cells	Natural Killer Cells
TGF-β	Transforming Growth Factor-Beta
HSCs	Hepatic Stellate Cells
APCs	Antigen-Presenting Cells
MDSCs	Myeloid-Derived Suppressor Cells
TCR	T-Cell Receptor
TANs	Tumor-Associated Neutrophils
NETs	Neutrophil Extracellular Traps
dHGP	Desmoplastic Histopathological Growth Pattern
TAMs	Tumor-Associated Macrophages
GVB	Gut Vascular Barrier
FMT	Fecal Microbiota Transplantation
TCGA	The Cancer Genome Atlas
scRNA-seq	Single-Cell RNA Sequencing
PT	Peritumoral
TF	Tumor Invasive Front
TC	Tumor Center
IL-10	Interleukin-10
TSCs	Tumor Slice Cultures
TILs	Tumor-Infiltrating Lymphocytes
MWA	Microwave Ablation
SOC	Standard-of-Care
TMB	Tumor Mutational Burden
TKIs	Tyrosine Kinase Inhibitors
BSC	Best Supportive Care
SITC	The Society for Immunotherapy of Cancer

## References

[1] Bray F., Laversanne M., Sung H., Ferlay J., Siegel R.L., Soerjomataram I., Jemal A. (2024). Global Cancer Statistics 2022: GLOBOCAN Estimates of Incidence and Mortality Worldwide for 36 Cancers in 185 Countries. CA Cancer J. Clin..

[2] Slesser A.a.P., Simillis C., Goldin R., Brown G., Mudan S., Tekkis P.P. (2013). A Meta-Analysis Comparing Simultaneous versus Delayed Resections in Patients with Synchronous Colorectal Liver Metastases. Surg. Oncol..

[3] Nordlinger B., Sorbye H., Glimelius B., Poston G.J., Schlag P.M., Rougier P., Bechstein W.O., Primrose J.N., Walpole E.T., Finch-Jones M. (2013). Perioperative FOLFOX4 Chemotherapy and Surgery versus Surgery Alone for Resectable Liver Metastases from Colorectal Cancer (EORTC 40983): Long-Term Results of a Randomised, Controlled, Phase 3 Trial. Lancet Oncol..

[4] Tsilimigras D.I., Brodt P., Clavien P.-A., Muschel R.J., D’Angelica M.I., Endo I., Parks R.W., Doyle M., de Santibañes E., Pawlik T.M. (2021). Liver Metastases. Nat. Rev. Dis. Primers.

[5] Grothey A., Van Cutsem E., Sobrero A., Siena S., Falcone A., Ychou M., Humblet Y., Bouché O., Mineur L., Barone C. (2013). Regorafenib Monotherapy for Previously Treated Metastatic Colorectal Cancer (CORRECT): An International, Multicentre, Randomised, Placebo-Controlled, Phase 3 Trial. Lancet.

[6] Saltz L.B., Clarke S., Díaz-Rubio E., Scheithauer W., Figer A., Wong R., Koski S., Lichinitser M., Yang T.-S., Rivera F. (2008). Bevacizumab in Combination with Oxaliplatin-Based Chemotherapy as First-Line Therapy in Metastatic Colorectal Cancer: A Randomized Phase III Study. J. Clin. Oncol..

[7] Folprecht G., Gruenberger T., Bechstein W.O., Raab H.-R., Lordick F., Hartmann J.T., Lang H., Frilling A., Stoehlmacher J., Weitz J. (2010). Tumour Response and Secondary Resectability of Colorectal Liver Metastases Following Neoadjuvant Chem-otherapy with Cetuximab: The CELIM Randomised Phase 2 Trial. Lancet Oncol..

[8] He W.-Z., Hu W.-M., Wang F., Rong Y.-M., Yang L., Xie Q.-K., Yang Y.-Z., Jiang C., Qiu H.-J., Lu J.-B. (2019). Comparison of Mismatch Repair Status Between Primary and Matched Metastatic Sites in Patients With Colo-rectal Cancer. J. Natl. Compr. Cancer Netw..

[9] Jung J., Kang Y., Lee Y.J., Kim E., Ahn B., Lee E., Kim J.Y., Lee J.H., Lee Y., Kim C.H. (2017). Comparison of the Mismatch Repair System between Primary and Metastatic Colorectal Cancers Using Im-munohistochemistry. J. Pathol. Transl. Med..

[10] Yu J., Green M.D., Li S., Sun Y., Journey S.N., Choi J.E., Rizvi S.M., Qin A., Waninger J.J., Lang X. (2021). Liver Metastasis Restrains Immunotherapy Efficacy via Macrophage-Mediated T Cell Elimination. Nat. Med..

[11] Johnson B., Haymaker C.L., Parra E.R., Soto L.M.S., Wang X., Thomas J.V., Dasari A., Morris V.K., Raghav K., Vilar E. (2022). Phase II Study of Durvalumab (Anti-PD-L1) and Trametinib (MEKi) in Microsatellite Stable (MSS) Metastatic Colorectal Cancer (mCRC). J. Immunother. Cancer.

[12] Fukuoka S., Hara H., Takahashi N., Kojima T., Kawazoe A., Asayama M., Yoshii T., Kotani D., Tamura H., Mikamoto Y. (2020). Regorafenib Plus Nivolumab in Patients With Advanced Gastric or Colorectal Cancer: An Open-Label, Dose-Escalation, and Dose-Expansion Phase Ib Trial (REGONIVO, EPOC1603). J. Clin. Oncol..

[13] Chen E.X., Loree J.M., Titmuss E., Jonker D.J., Kennecke H.F., Berry S., Couture F., Ahmad C.E., Goffin J.R., Kavan P. (2023). Liver Metastases and Immune Checkpoint Inhibitor Efficacy in Patients with Refractory Metastatic Colorectal Cancer: A Secondary Analysis of a Randomized Clinical Trial. JAMA Netw. Open.

[14] Yu S., Zhang S., Xu H., Yang G., Xu F., Yang L., Chen D., An G., Wang Y. (2023). Organ-Specific Immune Checkpoint Inhibitor Treatment in Lung Cancer: A Systematic Review and Meta-Analysis. BMJ Open.

[15] Galle P.R., Finn R.S., Qin S., Ikeda M., Zhu A.X., Kim T.-Y., Kudo M., Breder V., Merle P., Kaseb A. (2021). Patient-Reported Outcomes with Atezolizumab plus Bevacizumab versus Sorafenib in Patients with Unresectable Hepatocellular Carcinoma (IMbrave150): An Open-Label, Randomised, Phase 3 Trial. Lancet Oncol..

[16] Zeng X., Ward S.E., Zhou J., Cheng A.S.L. (2021). Liver Immune Microenvironment and Metastasis from Colorectal Cancer-Pathogenesis and Therapeutic Perspectives. Cancers.

[17] Wu K., Zhang G., Shen C., Zhu L., Yu C., Sartorius K., Ding W., Jiang Y., Lu Y. (2024). Role of T Cells in Liver Metastasis. Cell Death Dis..

[18] Van den Eynden G.G., Majeed A.W., Illemann M., Vermeulen P.B., Bird N.C., Høyer-Hansen G., Eefsen R.L., Reynolds A.R., Brodt P. (2013). The Multifaceted Role of the Microenvironment in Liver Metastasis: Biology and Clinical Implications. Cancer Res..

[19] Sun Y., Hu H., Liu Z., Xu J., Gao Y., Zhan X., Zhou S., Zhong W., Wu D., Wang P. (2023). Macrophage STING Signaling Promotes NK Cell to Suppress Colorectal Cancer Liver Metastasis via 4-1BBL/4-1BB Co-Stimulation. J. Immunother. Cancer.

[20] Wang Y., Zhong X., He X., Hu Z., Huang H., Chen J., Chen K., Zhao S., Wei P., Li D. (2023). Liver Metastasis from Colorectal Cancer: Pathogenetic Development, Immune Landscape of the Tumour Microenvironment and Therapeutic Approaches. J. Exp. Clin. Cancer Res..

[21] Keirsse J., Van Damme H., Geeraerts X., Beschin A., Raes G., Van Ginderachter J.A. (2018). The Role of Hepatic Macrophages in Liver Metastasis. Cell Immunol..

[22] Langhans B., Nischalke H.D., Krämer B., Dold L., Lutz P., Mohr R., Vogt A., Toma M., Eis-Hübinger A.M., Nattermann J. (2019). Role of Regulatory T Cells and Checkpoint Inhibition in Hepatocellular Carcinoma. Cancer Immunol. Immunother..

[23] Sieminska I., Baran J. (2020). Myeloid-Derived Suppressor Cells in Colorectal Cancer. Front. Immunol..

[24] Zhou H., Liu Z., Wang Y., Wen X., Amador E.H., Yuan L., Ran X., Xiong L., Ran Y., Chen W. (2022). Colorectal Liver Metastasis: Molecular Mechanism and Interventional Therapy. Signal Transduct. Target. Ther..

[25] Höppener D.J., Galjart B., Nierop P.M.H., Buisman F.E., van der Stok E.P., Coebergh van den Braak R.R.J., van Amerongen M.J., Balachandran V.P., Jarnagin W.R., Kingham T.P. (2021). Histopathological Growth Patterns and Survival After Resection of Colorectal Liver Metastasis: An External Validation Study. JNCI Cancer Spectr..

[26] Höppener D.J., Nierop P.M.H., Hof J., Sideras K., Zhou G., Visser L., Gouw A.S.H., de Jong K.P., Sprengers D., Kwekkeboom J. (2020). Enrichment of the Tumour Immune Microenvironment in Patients with Desmoplastic Colorectal Liver Metastasis. Br. J. Cancer.

[27] Wu Y., Yang S., Ma J., Chen Z., Song G., Rao D., Cheng Y., Huang S., Liu Y., Jiang S. (2022). Spatiotemporal Immune Landscape of Colorectal Cancer Liver Metastasis at Single-Cell Level. Cancer Discov..

[28] Sampaio-Ribeiro G., Ruivo A., Silva A., Santos A.L., Oliveira R.C., Gama J., Cipriano M.A., Tralhão J.G., Paiva A. (2023). Innate Immune Cells in the Tumor Microenvironment of Liver Metastasis from Colorectal Cancer: Contribution to a Comprehensive Therapy. Cancers.

[29] Lee J.C., Mehdizadeh S., Smith J., Young A., Mufazalov I.A., Mowery C.T., Daud A., Bluestone J.A. (2020). Regulatory T Cell Control of Systemic Immunity and Immunotherapy Response in Liver Metastasis. Sci. Immunol..

[30] Ho W.W., Gomes-Santos I.L., Aoki S., Datta M., Kawaguchi K., Talele N.P., Roberge S., Ren J., Liu H., Chen I.X. (2021). Dendritic Cell Paucity in Mismatch Repair–Proficient Colorectal Cancer Liver Metastases Limits Immune Checkpoint Blockade Efficacy. Proc. Natl. Acad. Sci. USA.

[31] Chen S., Zhang L., Li M., Zhang Y., Sun M., Wang L., Lin J., Cui Y., Chen Q., Jin C. (2022). Fusobacterium Nucleatum Reduces METTL3-Mediated m6A Modification and Contributes to Colorectal Cancer Metastasis. Nat. Commun..

[32] Bertocchi A., Carloni S., Ravenda P.S., Bertalot G., Spadoni I., Lo Cascio A., Gandini S., Lizier M., Braga D., Asnicar F. (2021). Gut Vascular Barrier Impairment Leads to Intestinal Bacteria Dissemination and Colorectal Cancer Metastasis to Liver. Cancer Cell.

[33] Xu C., Fan L., Lin Y., Shen W., Qi Y., Zhang Y., Chen Z., Wang L., Long Y., Hou T. (2021). Fusobacterium Nucleatum Promotes Colorectal Cancer Metastasis through miR-1322/CCL20 Axis and M2 Polarization. Gut Microbes.

[34] Mignini I., Piccirilli G., Galasso L., Termite F., Esposto G., Ainora M.E., Gasbarrini A., Zocco M.A. (2024). From the Colon to the Liver: How Gut Microbiota May Influence Colorectal Cancer Metastatic Potential. J. Clin. Med..

[35] Jiang S.-S., Xie Y.-L., Xiao X.-Y., Kang Z.-R., Lin X.-L., Zhang L., Li C.-S., Qian Y., Xu P.-P., Leng X.-X. (2023). Fusobacterium Nucleatum-Derived Succinic Acid Induces Tumor Resistance to Immunotherapy in Colorectal Cancer. Cell Host Microbe.

[36] Zhao W., Lei J., Ke S., Chen Y., Xiao J., Tang Z., Wang L., Ren Y., Alnaggar M., Qiu H. (2023). Fecal Microbiota Transplantation plus Tislelizumab and Fruquintinib in Refractory Microsatellite Stable Metastatic Colorectal Cancer: An Open-Label, Single-Arm, Phase II Trial (RENMIN-215). EClinicalMedicine.

[37] Liu Y., Zhang Q., Xing B., Luo N., Gao R., Yu K., Hu X., Bu Z., Peng J., Ren X. (2022). Immune Phenotypic Linkage between Colorectal Cancer and Liver Metastasis. Cancer Cell.

[38] Sathe A., Mason K., Grimes S.M., Zhou Z., Lau B.T., Bai X., Su A., Tan X., Lee H., Suarez C.J. (2023). Colorectal Cancer Metastases in the Liver Establish Immunosuppressive Spatial Networking between Tu-mor-Associated SPP1+ Macrophages and Fibroblasts. Clin. Cancer Res..

[39] He Y., Han Y., Fan A., Li D., Wang B., Ji K., Wang X., Zhao X., Lu Y. (2022). Multi-Perspective Comparison of the Immune Microenvironment of Primary Colorectal Cancer and Liver Metastases. J. Transl. Med..

[40] Zhou S.-N., Pan W.-T., Pan M.-X., Luo Q.-Y., Zhang L., Lin J.-Z., Zhao Y.-J., Yan X.-L., Yuan L.-P., Zhang Y.-X. (2021). Comparison of Immune Microenvironment Between Colon and Liver Metastatic Tissue in Colon Cancer Patients with Liver Metastasis. Dig. Dis. Sci..

[41] Ye J., Guo W., Wang C., Egelston C.A., D’Apuzzo M., Shankar G., Fakih M.G., Lee P.P. (2023). Peritumoral Immune-Suppressive Mechanisms Impede Intratumoral Lymphocyte Infiltration into Colorectal Cancer Liver versus Lung Metastases. Cancer Res. Commun..

[42] Deng J.-Y., Gou Q., Yang L., Chen Z.-H., Yang M.-Y., Yang X.-R., Yan H.-H., Wei X.-W., Liu J.-Q., Su J. (2023). Immune Suppressive Microenvironment in Liver Metastases Contributes to Organ-Specific Response of Immunotherapy in Advanced Non-Small Cell Lung Cancer. J. Immunother. Cancer.

[43] Sullivan K.M., Jiang X., Guha P., Lausted C., Carter J.A., Hsu C., Labadie K.P., Kohli K., Kenerson H.L., Daniel S.K. (2023). Blockade of Interleukin 10 Potentiates Antitumour Immune Function in Human Colorectal Cancer Liver Metastases. Gut.

[44] Shao D., Chen Y., Huang H., Liu Y., Chen J., Zhu D., Zheng X., Chen L., Jiang J. (2022). LAG3 Blockade Coordinates with Microwave Ablation to Promote CD8^+^ T Cell-Mediated Anti-Tumor Immunity. J. Transl. Med..

[45] Ramadori P., Gallage S., Heikenwälder M.F. (2023). Unique Tumour Microenvironment: When Ferroptosis Activation Boosts ICI of Liver Cancer. Gut.

[46] Conche C., Finkelmeier F., Pešić M., Nicolas A.M., Böttger T.W., Kennel K.B., Denk D., Ceteci F., Mohs K., Engel E. (2023). Combining Ferroptosis Induction with MDSC Blockade Renders Primary Tumours and Metastases in Liver Sensitive to Immune Checkpoint Blockade. Gut.

[47] Lenz H.-J., Parikh A., Spigel D.R., Cohn A.L., Yoshino T., Kochenderfer M., Elez E., Shao S.H., Deming D., Holdridge R. (2024). Modified FOLFOX6 plus Bevacizumab with and without Nivolumab for First-Line Treatment of Metastatic Colorectal Cancer: Phase 2 Results from the CheckMate 9X8 Randomized Clinical Trial. J. Immunother. Cancer.

[48] Upfront FOLFOXIRI Plus Bevacizumab with or Without Atezolizumab in the Treatment of Patients with Metastatic Colorectal Cancer (AtezoTRIBE): A Multicentre, Open-Label, Randomised, Controlled, Phase 2 Trial-PubMed. https://pubmed.ncbi.nlm.nih.gov/35636444/.

[49] Effect of Combined Immune Checkpoint Inhibition vs Best Supportive Care Alone in Patients with Advanced Colorectal Cancer: The Canadian Cancer Trials Group CO.26 Study|Cancer Biomarkers|JAMA Oncology|JAMA Network. https://jamanetwork.com/journals/jamaoncology/fullarticle/2765332.

[50] Diaz L.A., Shiu K.-K., Kim T.-W., Jensen B.V., Jensen L.H., Punt C., Smith D., Garcia-Carbonero R., Benavides M., Gibbs P. (2022). Pembrolizumab versus Chemotherapy for Microsatellite Instability-High or Mismatch Repair-Deficient Metastatic Colorectal Cancer (KEYNOTE-177): Final Analysis of a Randomised, Open-Label, Phase 3 Study. Lancet Oncol..

[51] Lenz H.-J., Cutsem E.V., Limon M.L., Wong K.Y.M., Hendlisz A., Aglietta M., García-Alfonso P., Neyns B., Luppi G., Cardin D.B. (2021). First-Line Nivolumab Plus Low-Dose Ipilimumab for Microsatellite Instability-High/Mismatch Repair-Deficient Metastatic Colorectal Cancer: The Phase II CheckMate 142 Study. J. Clin. Oncol..

[52] Manca P., Corti F., Intini R., Mazzoli G., Miceli R., Germani M.M., Bergamo F., Ambrosini M., Cristarella E., Cerantola R. (2023). Tumour Mutational Burden as a Biomarker in Patients with Mismatch Repair Deficient/Microsatellite Instability-High Metastatic Colorectal Cancer Treated with Immune Checkpoint Inhibitors. Eur. J. Cancer.

[53] Wang F., Peng J., Liang X., Cheng Y., Deng Y., Chen K., Zhang M., Zhang J., Wang W., Cao B. (2024). First-Line Serplulimab plus HLX04 and XELOX versus Placebo plus Bevacizumab and XELOX in Metastatic Colorectal Cancer: A Phase 2/3 Study. J. Clin. Oncol..

[54] Wang F., Wang Z.-X., Peng J., Liang X., Cheng Y., Deng Y., Chen K., Zhang M., Zhang J., Wang W. (2024). First-Line Serplulimab plus HLX04 and XELOX versus Placebo plus Bevacizumab and XELOX in Metastatic Colorectal Cancer: A Phase 2/3 Study. J. Clin. Oncol..

[55] First-Line Durvalumab and Tremelimumab with Chemotherapy in RAS-Mutated Metastatic Colorectal Cancer: A Phase 1b/2 Trial-PubMed. https://pubmed.ncbi.nlm.nih.gov/37563240/.

[56] Kawazoe A., Xu R.-H., García-Alfonso P., Passhak M., Teng H.-W., Shergill A., Gumus M., Qvortrup C., Stintzing S., Towns K. (2024). Lenvatinib Plus Pembrolizumab Versus Standard of Care for Previously Treated Metastatic Colorectal Cancer: Final Analysis of the Randomized, Open-Label, Phase III LEAP-017 Study. J. Clin. Oncol..

[57] Fakih M., Sandhu J., Lim D., Li X., Li S., Wang C. (2023). Regorafenib, Ipilimumab, and Nivolumab for Patients With Microsatellite Stable Colorectal Cancer and Dis-ease Progression With Prior Chemotherapy: A Phase 1 Nonrandomized Clinical Trial. JAMA Oncol..

[58] Zhao Y., Ma Y., Zang A., Cheng Y., Zhang Y., Wang X., Chen Z., Qu S., He J., Chen C. (2023). First-in-Human Phase I/Ib Study of QL1706 (PSB205), a Bifunctional PD1/CTLA4 Dual Blocker, in Patients with Advanced Solid Tumors. J. Hematol. Oncol..

[59] Wang F., Jin Y., Wang M., Luo H.-Y., Fang W.-J., Wang Y.-N., Chen Y.-X., Huang R.-J., Guan W.-L., Li J.-B. (2024). Combined Anti-PD-1, HDAC Inhibitor and Anti-VEGF for MSS/pMMR Colorectal Cancer: A Randomized Phase 2 Trial. Nat. Med..

[60] Pagès F., Mlecnik B., Marliot F., Bindea G., Ou F.-S., Bifulco C., Lugli A., Zlobec I., Rau T.T., Berger M.D. (2018). International Validation of the Consensus Immunoscore for the Classification of Colon Cancer: A Prognostic and Accuracy Study. Lancet.

[61] Argilés G., Tabernero J., Labianca R., Hochhauser D., Salazar R., Iveson T., Laurent-Puig P., Quirke P., Yoshino T., Taieb J. (2020). Localised Colon Cancer: ESMO Clinical Practice Guidelines for Diagnosis, Treatment and Follow-Up†. Ann. Oncol..

[62] Antoniotti C., Rossini D., Pietrantonio F., Salvatore L., Lonardi S., Tamberi S., Marmorino F., Moretto R., Prisciandaro M., Tamburini E. (2024). Upfront Fluorouracil, Leucovorin, Oxaliplatin, and Irinotecan Plus Bevacizumab with or Without Atezoli-zumab for Patients with Metastatic Colorectal Cancer: Updated and Overall Survival Results of the ATEZOTRIBE Study. J. Clin. Oncol..

